# Obesity and Natural Products: Mechanisms, Therapeutic Potential, and Future Directions

**DOI:** 10.1002/fsn3.71575

**Published:** 2026-03-11

**Authors:** Ohoud M. Marie, Abdelghafar Abu‐Elsaoud

**Affiliations:** ^1^ Chemistry Department, Faculty of Science Suez Canal University Ismailia Egypt; ^2^ Department of Biology, College of Science Imam Mohammad Ibn Saud Islamic University (IMSIU) Riyadh Saudi Arabia

**Keywords:** adipogenesis, antioxidants, functional foods, insulin resistance, metabolic syndrome, natural products, obesity, phytochemicals

## Abstract

Obesity represents a major global public health challenge, currently affecting over 890 million adults worldwide and substantially increasing the risk of type 2 diabetes, cardiovascular diseases, and metabolic syndrome. Although several pharmacological agents are available for obesity management, their clinical use is often limited by adverse effects, high costs, and suboptimal long‐term efficacy. These limitations have driven growing interest in natural products as complementary or alternative therapeutic options. Bioactive compounds derived from medicinal plants, fruits, and vegetables, such as polyphenols, flavonoids, alkaloids, terpenoids, saponins, and carotenoids, have shown promising anti‐obesity effects through multi‐targeted mechanisms. This narrative review summarizes current in vitro, preclinical, and clinical evidence on the role of natural products in obesity management, with particular emphasis on their ability to modulate lipid metabolism, inhibit adipogenesis, enhance energy expenditure, regulate appetite‐related pathways, influence gut microbiota, and attenuate oxidative stress and inflammation. Compounds including epigallocatechin‐3‐gallate, curcumin, resveratrol, capsaicin, and berberine have demonstrated notable efficacy in experimental models, with increasing clinical data supporting their therapeutic potential. Nonetheless, challenges related to standardization, bioavailability, quality control, and regulatory approval remain major barriers to clinical translation. Addressing these issues is essential for the rational integration of natural products into evidence‐based obesity management strategies.

## Graphical abstract

Natural products such as EGCG, berberine, curcumin, and capsaicin modulate key metabolic pathways including AMPK activation, adipogenesis inhibition, and inflammatory signaling. While clinical trials demonstrate modest benefits on body weight and metabolic parameters, translational challenges related to bioavailability, dose feasibility, and regulatory variability remain critical considerations.

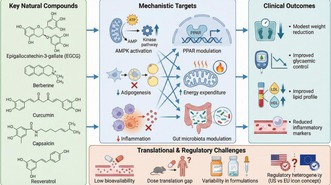

## 1 Introduction

### 1.1 The Global Obesity Epidemic

Obesity represents one of the most pressing public health challenges of the 21st century, characterized by excessive accumulation of adipose tissue that impairs health and quality of life (Ahmed and Mohammed 2025; Lewandowska et al. 2025). According to the World Health Organization (WHO), global obesity prevalence has tripled since 1975, with more than 2.5 billion adults classified as overweight or obese in 2022 (Ullah and Tamanna 2025). The World Obesity Atlas 2025 projects that by 2035, over 1.13 billion adults will be living with obesity, representing a 115% increase from 2010 levels (Tareke et al. 2025). This alarming trajectory is observed across both developed and developing nations, with particularly rapid increases in low‐and middle‐income countries (Koliaki et al. 2023).

The obesity epidemic extends beyond adults to affect pediatric populations, with 160 million children and adolescents suffering from obesity globally in 2022, an eightfold increase since 1990 (Wang and Lim 2012; Zhang, Liu, et al. 2024). The economic burden of obesity is staggering, with projected costs reaching $4 trillion USD by 2035, equivalent to approximately 3% of global GDP (Wang and Lim 2012).

### 1.2 Scope and Literature Selection

This article is a narrative review intended to provide an overview of the role of natural products in obesity management. Relevant literature was identified through targeted searches of PubMed, Web of Science, and Scopus up to 2025, using keywords related to obesity, natural products, phytochemicals, and clinical studies. Priority was given to mechanistically informative experimental studies, well‐conducted clinical trials, and recent meta‐analyses published in peer‐reviewed journals. This review does not aim to follow a systematic review protocol, and study selection was based on relevance and scientific rigor rather than exhaustive inclusion. Where appropriate, methodological quality and risk‐of‐bias considerations reported in the original studies and meta‐analyses were discussed qualitatively.

### 1.3 Pathophysiology of Obesity

Obesity is a complex, multifactorial disease involving intricate interactions between genetic predisposition, environmental factors, behavioral patterns, and metabolic dysregulation (Ahmed and Mohammed 2025; Młynarska et al. 2025).

#### 1.3.1 Adipogenesis and Adipocyte Differentiation

Adipogenesis, the differentiation of mesenchymal stem cell‐derived preadipocytes into mature, lipid‐storing adipocytes, constitutes a critical process in obesity pathogenesis (Ali et al. 2013). This complex developmental program is orchestrated by hierarchical transcriptional cascades initiated by growth factors (particularly insulin) and mediated by key transcription factors including peroxisome proliferator‐activated receptor gamma (PPARγ) and CCAAT/enhancer‐binding proteins (C/EBPα and C/EBPβ). PPARγ serves as the master regulator of adipogenesis, with its activation being both necessary and sufficient for driving adipocyte terminal differentiation (Madsen et al. 2014). Dysregulated adipogenesis in obesity involves excessive preadipocyte recruitment and differentiation, leading to increased adipocyte number (hyperplasia) and hypertrophy. Understanding these mechanisms is crucial because natural compounds demonstrating PPARγ antagonism or selective modulation represent potential intervention points in the adipogenic cascade (Richard et al. 2020).

#### 1.3.2 Dysfunctional Lipid Metabolism and Lipogenesis Dysregulation

Obesity is fundamentally characterized by a disrupted balance between lipid anabolism (synthesis) and catabolism (mobilization) (Ludwig et al. 2022). De novo lipogenesis, the biosynthetic pathway converting carbohydrates to fatty acids, is profoundly dysregulated in obesity. Acetyl‐CoA carboxylase (ACC) functions as the rate‐limiting enzyme in de novo fatty acid synthesis, catalyzing the conversion of acetyl‐CoA into malonyl‐CoA, the key committed intermediate utilized by fatty acid synthase (FAS) for chain elongation. The transcription factor sterol regulatory element‐binding protein‐1c (SREBP‐1c) acts as the principal regulator of lipogenesis, orchestrating the coordinated expression of genes encoding lipogenic enzymes (Zu et al. 2013).

Conversely, the mobilization and catabolism of stored triglycerides (lipolysis) are substantially impaired in obesity. Hormone‐sensitive lipase (HSL), adipose triglyceride lipase (ATGL), and monoacylglycerol lipase constitute the critical enzymes governing triglyceride hydrolysis into free fatty acids and glycerol (Morak et al. 2012). In obesity, lipolytic capacity is suppressed through multiple mechanisms including impaired sympathetic nervous system signaling and reduced expression of lipolytic enzymes (Thorp and Schlaich 2015).

#### 1.3.3 Oxidative Stress, Chronic Inflammation, and Adipose Tissue Dysfunction

Adipose tissue in obesity exhibits elevated production of reactive oxygen species (ROS) through NADPH oxidases, mitochondrial dysfunction, and inflammatory cell infiltration (DeVallance et al. 2019; Lefranc et al. 2018; Olivares‐Vicente and Herranz‐López 2025). ROS activate stress‐sensitive kinases (JNK, IKKβ) and transcription factors (NF‐κB) that amplify inflammatory cytokine production and impair insulin signaling (Olivares‐Vicente and Herranz‐López 2025; Zhang et al. 2016).

Elevated ROS levels directly damage cellular lipids, proteins, and nucleic acids, simultaneously activating redox‐sensitive transcription factors that upregulate inflammatory gene expression (Bellanti et al. 2025). These pathological processes play a central role in the development of insulin resistance, impairment of endothelial function, and the progression of various metabolic disorders (Manna and Jain 2015).

Obesity simultaneously manifests as a state of chronic, low‐grade systemic inflammation characterized by elevated circulating levels of pro‐inflammatory cytokines (tumor necrosis factor‐alpha, interleukin‐6, interleukin‐1beta). This inflammatory state originates from multiple cellular sources, particularly hypertrophic adipocytes themselves, which shift toward enhanced secretion of pro‐inflammatory adipokines. The chronic inflammatory milieu drives insulin resistance, impairs metabolic flexibility, and promotes development of obesity‐related comorbidities (Gkrinia and Belančić 2025).

#### 1.3.4 Hormonal Dysregulation and Neuroendocrine Dysfunction

Obesity involves profound dysregulation of multiple hormonal axes governing energy homeostasis (Kong, Yang, Nie, et al. 2025). Leptin, the principal anorexigenic hormone secreted by adipocytes, exhibits paradoxically elevated circulating levels in obesity despite hyperphagia, a phenomenon termed leptin resistance (Hu et al. 2025). At the molecular level, obesity‐associated chronic inflammation and elevated circulating free fatty acids impair leptin signaling through the hypothalamic leptin receptor (Jais and Brüning 2017).

Ghrelin, the orexigenic “hunger hormone” secreted primarily by gastric cells, shows dysregulated secretory patterns in obesity, with impaired postprandial suppression contributing to sustained appetite (Müller et al. 2015). Additional hormonal disturbances central to obesity pathophysiology include insulin resistance and compensatory hyperinsulinemia, impaired glucagon‐like peptide‐1 (GLP‐1) secretion and signaling, and altered glucose‐dependent insulinotropic polypeptide (GIP) signaling (Müller et al. 2019).

### 1.4 Limitations of Current Pharmacological Treatments

Despite significant research efforts, the approved anti‐obesity medications remain limited, and existing drugs are associated with substantial limitations (Zahran et al. 2025). Currently approved pharmacotherapies include orlistat (lipase inhibitor), phentermine/topiramate (appetite suppressant/anticonvulsant combination), naltrexone/bupropion (opioid antagonist/antidepressant combination), liraglutide and semaglutide (glucagon‐like peptide‐1 receptor agonists), and tirzepatide (dual GLP‐1/GIP receptor agonist) (Fredrick et al. 2025; Jordan et al. 2024).

Orlistat, the only non‐systemically absorbed anti‐obesity drug, produces modest weight loss (2.9% placebo‐subtracted) but is limited by gastrointestinal side effects including steatorrhea, fecal incontinence, and malabsorption of fat‐soluble vitamins (Heck et al. 2000; Uuh Narvaez and Chan Zapata 2025). Phentermine‐based combinations carry cardiovascular risks and are contraindicated in patients with hypertension, cardiovascular disease, or hyperthyroidism (Jordan et al. 2014). The GLP‐1 receptor agonists, while more effective (5%–15% weight loss), are associated with nausea, vomiting, diarrhea, constipation, and potential risks of pancreatitis and gallbladder disease. Additionally, these medications are expensive, require long‐term administration, and weight regain commonly occurs upon discontinuation (Filippatos et al. 2014; Mehta et al. 2025).

Furthermore, anti‐obesity drugs demonstrate variable efficacy across populations, with inadequate inclusion of racial and ethnic minorities in clinical trials limiting generalizability (Bomberg et al. 2022; Gadde and Atkins 2020). Approximately 30%–40% of patients fail to achieve clinically meaningful weight loss (≥ 5% body weight) with current pharmacotherapies. These limitations underscore the critical need for alternate therapeutic protocols with improved efficiency, safety, and accessibility (Gadde and Atkins 2020).

### 1.5 Rationale for Natural Products in Obesity Management

Natural products derived from medicinal plants, fruits, vegetables, and other natural sources have been utilized for millennia in traditional medicine systems worldwide (Chaachouay and Zidane 2024; Theodoridis et al. 2023), including Traditional Chinese Medicine (TCM), Ayurveda, and folk medicine traditions. The therapeutic potential of phytochemicals for obesity management has garnered substantial scientific interest due to several compelling advantages: (1) multi‐targeted mechanisms of action addressing the complex, multifactorial nature of obesity; (2) generally favorable safety profiles with lower incidence of severe adverse effects compared to synthetic drugs; (3) relatively low cost and widespread accessibility; (4) potential synergistic effects when used in combination; and (5) alignment with consumer preferences for natural health products (Bhardwaj et al. 2021; Rao 2025; Saad et al. 2022).

Epidemiological studies have consistently demonstrated inverse associations between consumption of plant‐based diets rich in bioactive phytochemicals and obesity prevalence (Hossain and Wazed 2025). Mechanistic investigations have revealed that natural compounds modulate multiple pathways involved in energy homeostasis, adipose tissue biology, inflammation, and oxidative stress (Liu, Liu, et al. 2025). These compounds include diverse chemical classes such as polyphenols (flavonoids, phenolic acids, stilbenes, lignans), alkaloids, terpenoids, saponins, and carotenoids, each exhibiting distinct yet potentially complementary mechanisms (Kumar and Nirmal 2023; Riaz et al. 2023).

This comprehensive review critically evaluates the current state of knowledge regarding natural products in obesity management, synthesizing evidence from in vitro, preclinical, as well as clinical studies. We examine molecular mechanisms of action, discuss notable bioactive compounds and their botanical sources, analyze safety and standardization challenges, and propose future directions for research and clinical application.

## 2 Role of Natural Products in Obesity Management

### 2.1 Historical Context and Traditional Uses

The use of medicinal plants for weight management has deep historical roots spanning multiple civilizations and medical traditions (Chaachouay and Zidane 2024; De Freitas Junior and Almeida Jr 2017). Traditional Chinese Medicine has employed herbal formulations containing *Rheum palmatum* (rhubarb), *Citrus aurantium* (bitter orange), and *Nelumbo nucifera* (lotus leaf) for treating conditions related to excess phlegm and dampness, which correlate with modern obesity (Shao et al. 2022; Wen et al. 2024). Ayurvedic medicine has utilized *Gymnema sylvestre* , Commiphora mukul (guggul), and Garcinia cambogia for metabolic disorders and weight control (Pandey et al. 2013). Indigenous populations worldwide have incorporated appetite‐suppressant and thermogenic plants into their pharmacopeias, providing a rich foundation for contemporary research (Upton et al. 2024).

### 2.2 Evidence‐Based Studies on Plant Extracts and Phytochemicals

Several systematic reviews and meta‐analyses have investigated the therapeutic potential of different plant‐derived extracts and bioactive compounds in the prevention and management of obesity. A comprehensive meta‐analysis examining 279 clinical trials found that herbal medicines containing green tea, *Phaseolus vulgaris* (white kidney bean), Garcinia cambogia, and *Nigella sativa* demonstrated significant anti‐obesity effects with standardized mean differences ranging from 0.3 to 0.8 for anthropometric outcomes (Mamun and Rakib 2024). Green tea extracts rich in catechins, particularly EGCG, have been most extensively studied, with clinical trials demonstrating 1.5–3 kg greater weight loss compared to placebo over 12 weeks (Hu et al. 2018; Jurgens et al. 2012).

Preclinical studies using high‐fat diet (HFD)‐induced obesity models have provided compelling evidence for the anti‐obesity potential of numerous plant extracts. *Camellia sinensis* (green tea), *Curcuma longa* (turmeric), Garcinia cambogia, Irvingia gabonensis (African mango), and *Morus alba* (white mulberry) have demonstrated significant reductions in body weight, fat mass, and metabolic parameters in rodent models (Aziz et al. 2023; Kim et al. 2021). These effects are mediated through multiple mechanisms including inhibition of pancreatic lipase, reduction of dietary fat absorption, modulation of adipose tissue metabolism, and regulation of appetite‐controlling hormones (Subramaniyan and Hanim 2025).

### 2.3 Bioactive Compounds and Their Distribution

Natural products contain diverse arrays of bioactive phytochemicals responsible for their anti‐obesity effects (Kumar and Nirmal 2023). Polyphenols represent the most extensively studied class, comprising flavonoids (e.g., quercetin, catechins, anthocyanins), phenolic acids (e.g., chlorogenic acid, caffeic acid), stilbenes (e.g., resveratrol), and lignans (Rudrapal and Rakshit 2024). These compounds are abundant in fruits (berries, grapes, and citrus), vegetables (onions, broccoli), tea, coffee, cocoa, and red wine (Tabolacci et al. 2023). Alkaloids, nitrogen‐containing compounds with potent biological activities, include capsaicin from chili peppers, berberine from Berberis species, and caffeine from coffee and tea (Cárdenas and Mojica 2025). Terpenoids represent a broad and structurally diverse class of naturally occurring compounds biosynthesized from repeating isoprene units, include carotenoids (β‐carotene, lycopene, fucoxanthin), triterpenes, and diterpenoids found in colorful fruits, vegetables, and seaweeds (Câmara and Perestrelo 2024). Saponins, glycosidic compounds with soap‐like properties, are present in legumes, ginseng, and quinoa, exhibiting lipase inhibition and metabolic regulation (Mieres‐Castro and Mora‐Poblete 2023).

The chemical diversity of these compounds underlies their multi‐targeted mechanisms, enabling simultaneous modulation of adipogenesis, lipid metabolism, inflammation, oxidative stress, and energy expenditure. This polypharmacological approach may prove superior to single‐target synthetic drugs for managing the complex pathophysiology of obesity (Makhoba and Viegas 2020; Mamun and Rakib 2024).

## 3 Mechanisms of Action

While natural products are often described as “multi‐targeted” agents, excessive mechanistic breadth may obscure clinically actionable pathways. Therefore, this review prioritizes mechanisms supported by human or late translational evidence, particularly those related to energy metabolism, insulin signaling, adipose inflammation, and lipid handling. Mechanisms identified exclusively in in‐vitro or early animal studies are briefly acknowledged but interpreted cautiously, as their contribution to therapeutic efficacy in humans remains uncertain.

### 3.1 Lipid Metabolism Regulation

**FIGURE 1:**
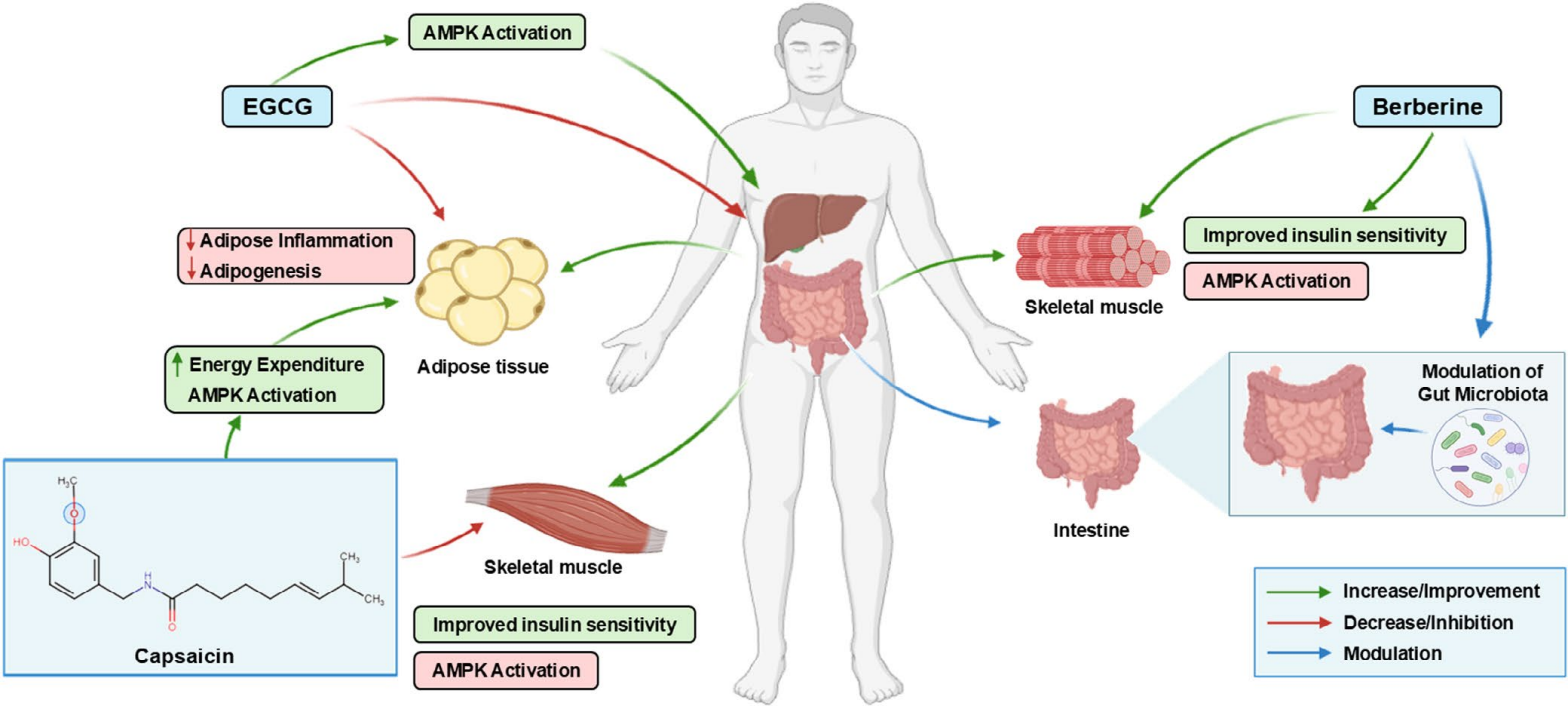
Multi‐targeted molecular mechanisms of selected natural products in obesity management.

Natural products exert profound effects on lipid metabolism through multiple complementary mechanisms (Cheng et al. 2020), as shown in Figure 1. At the intestinal level, certain compounds inhibit pancreatic lipase, the rate‐limiting enzyme for dietary triglyceride hydrolysis, thereby reducing fat absorption (Liu et al. 2020). Polyphenol‐rich extracts from *Camellia sinensis* , *Punica granatum* (pomegranate), and *Curcuma longa* demonstrate potent lipase inhibitory activity compared to orlistat in vitro (Kamran Javed 2024). EGCG interferes with lipid emulsification, digestion, and micellar solubilization, critical steps in intestinal fat absorption. Clinical studies have demonstrated increased fecal lipid excretion following green tea catechin supplementation, supporting this mechanism (Koo and Noh 2007).

Within adipose tissue, natural compounds modulate lipogenesis (fat synthesis) and lipolysis (fat breakdown) through transcriptional and post‐translational mechanisms (Nematbakhsh et al. 2021). Key transcription factors regulating adipogenesis include peroxisome proliferator‐activated receptor gamma (PPARγ), CCAAT/enhancer‐binding proteins (C/EBPα, C/EBPβ, C/EBPδ), and sterol regulatory element‐binding protein‐1c (SREBP‐1c). Many phytochemicals, including EGCG, curcumin, resveratrol, and genistein, downregulate these adipogenic transcription factors, thereby inhibiting preadipocyte differentiation and reducing adipocyte size (Madsen et al. 2014).

Berberine activates AMP‐activated protein kinase (AMPK), a master metabolic regulator that phosphorylates and inhibits acetyl‐CoA carboxylase (ACC), the rate‐limiting enzyme in fatty acid synthesis (Grahame Hardie 2014). AMPK activation also stimulates fatty acid oxidation by upregulating carnitine palmitoyltransferase‐1 (CPT‐1), facilitating mitochondrial fatty acid uptake and β‐oxidation. EGCG similarly activates AMPK in adipose tissue, liver, and skeletal muscle, promoting a shift from lipid storage to oxidation (Choi et al. 2024; Li, Ding, et al. 2025).

### 3.2 Adipogenesis Inhibition

Adipogenesis, the differentiation of preadipocytes into mature adipocytes, represents a critical process in adipose tissue expansion (Moreno‐Navarrete and Fernández‐Real 2017). This complex cascade is orchestrated by sequential activation of transcription factors, beginning with C/EBPβ and C/EBPδ, which induce expression of PPARγ and C/EBPα, the master regulators of terminal adipocyte differentiation (Fiedler et al. 2025; Guo et al. 2015). PPARγ activation triggers expression of downstream genes encoding adipocyte‐specific proteins including fatty acid‐binding protein 4 (FABP4, also known as aP2), fatty acid synthase (FAS), adiponectin, and perilipin (Abou Azar 2025; Preciado‐Ortiz et al. 2025).

Numerous natural compounds inhibit adipogenesis at multiple stages. Resveratrol suppresses early adipogenic events by activating sirtuin 1 (SIRT1), an NAD^+^‐dependent deacetylase that inhibits PPARγ activity and promotes fat mobilization (Costa Cdos et al. 2011). Quercetin and kaempferol downregulate C/EBPα and PPARγ expression while upregulating preadipocyte factor‐1 (Pref‐1), a negative regulator of adipogenesis. Curcumin inhibits adipogenesis through multiple pathways, including suppression of Wnt/β‐catenin signaling, which is critical for maintaining preadipocytes in an undifferentiated state (He et al. 2024; Perdicaro et al. 2020).

In 3T3‐L1 preadipocytes, a widely used in vitro model, EGCG profoundly inhibits terminal differentiation and lipid droplet formation, accompanied by reduced PPARγ, C/EBPα, and FAS expression (Aranaz et al. 2019; Wang et al. 2024). This effect is mediated through AMPK activation and inhibition of autophagy during early‐stage differentiation. Berberine similarly suppresses PPARγ and C/EBPα in adipocytes while promoting browning of white adipose tissue through AMPK‐dependent mechanisms (Ahmad et al. 2020; Khan et al. 2022).

### 3.3 Energy Expenditure Enhancement

Increasing energy expenditure through thermogenesis represents an attractive strategy for obesity management (Soliman et al. 2025). Brown adipose tissue (BAT) specializes in adaptive thermogenesis through uncoupling protein 1 (UCP1), which dissipates the mitochondrial proton gradient as heat rather than ATP synthesis. Additionally, certain white adipocytes can acquire brown‐like characteristics (termed “beige” or “brite” adipocytes) through a process called browning or beiging, contributing to enhanced energy expenditure (van der Vaart et al. 2021; Zhang et al. 2025).

Several natural compounds promote thermogenesis and browning of white adipose tissue. Capsaicin, the pungent principle of chili peppers, activates transient receptor potential vanilloid 1 (TRPV1) channels, triggering a thermogenic cascade involving calcium influx, AMPK activation, and upregulation of UCP1, PGC‐1α (PPARγ coactivator‐1α), and PRDM16 (PR domain containing 16), master regulators of brown adipocyte development (Fan et al. 2019). In HFD‐fed mice, dietary capsaicin induces browning of subcutaneous white adipose tissue, increases UCP1 expression, and enhances oxygen consumption, resulting in reduced body weight and improved metabolic parameters (Baskaran et al. 2016; Li, Chu, and Yang 2025).

Fucoxanthin, a marine carotenoid from brown seaweeds, stimulates UCP1 expression in white adipose tissue and increases metabolic rate in rodent models. Resveratrol promotes WAT browning through activation of SIRT1 and AMPK, upregulating PGC‐1α and mitochondrial biogenesis (Maeda et al. 2005; Peng et al. 2011).

Green tea catechins increase energy expenditure through multiple mechanisms, including inhibition of catechol‐O‐methyltransferase (COMT), which degrades norepinephrine, thereby prolonging sympathetic nervous system activation of thermogenesis (Diepvens et al. 2007; Sousa‐Filho et al. 2020).

### 3.4 Gut Microbiota Modulation

The gut microbiota plays a pivotal role in energy homeostasis, with obesity associated with distinct microbial signatures characterized by reduced diversity, altered Firmicutes‐to‐Bacteroidetes ratios, and functional changes in metabolic capacity (Magne et al. 2020). Dysbiotic gut microbiota contributes to obesity through multiple mechanisms: (1) increased energy harvest from diet, (2) modulation of gut permeability and metabolic endotoxemia, (3) altered bile acid metabolism, and (4) production of bioactive metabolites such as short‐chain fatty acids (SCFAs) (Breton and Galmiche 2022; Dahiya et al. 2017).

Natural products can beneficially modulate gut microbiota composition and function. Polyphenols, particularly those that are poorly absorbed in the small intestine, reach the colon where they are metabolized by gut bacteria, producing bioactive metabolites while simultaneously influencing microbial community structure (Wang et al. 2022). Green tea catechins increase the abundance of beneficial bacteria including *Akkermansia muciniphila* , Bifidobacterium, and Lactobacillus species while reducing potentially pathogenic Proteobacteria (Pérez‐Burillo et al. 2021; Yang et al. 2025).

Berberine modulates gut microbiota in HFD‐induced obese mice, reducing the Firmicutes‐to‐Bacteroidetes ratio and increasing SCFA‐producing bacteria (Yang et al. 2023). These changes correlate with improved glucose tolerance, reduced hepatic steatosis, and decreased inflammation. The gut microbiota‐dependent effects of berberine include enhanced intestinal barrier integrity, reduced lipopolysaccharide (LPS) translocation, and attenuation of metabolic endotoxemia (Kong, Yang, Nie, Zhang, et al. 2025; Zhang, Wu, et al. 2020).

### 3.5 Appetite Regulation

Appetite control involves complex neuroendocrine networks, primarily centered in the hypothalamus, which integrate peripheral signals from adipose tissue, pancreas, and gastrointestinal tract. Key hormones regulating appetite include leptin (anorexigenic), ghrelin (orexigenic), insulin, peptide YY (PYY), cholecystokinin (CCK), and glucagon‐like peptide‐1 (GLP‐1) (Abou‐Samra and Venema 2022; Blanco et al. 2017; Rønnestad et al. 2017). In obesity, leptin resistance develops, characterized by elevated circulating leptin levels but impaired hypothalamic signaling, contributing to persistent hyperphagia (Obradovic et al. 2021).

Natural products modulate appetite through multiple mechanisms. *Hoodia gordonii* extracts, traditionally used by San people of Southern Africa for appetite suppression, contain steroidal glycosides that activate hypothalamic ATP‐sensitive potassium channels, mimicking glucose‐induced satiety signals (Avula et al. 2006; Le Nevé et al. 2010). Garcinia cambogia, rich in hydroxy citric acid (HCA), inhibits ATP citrate lyase, reducing malonyl‐CoA availability for fatty acid synthesis while simultaneously increasing serotonin synthesis, promoting satiety (Andueza et al. 2021).

EGCG influences appetite regulation by modulating ghrelin and adiponectin levels (Fernandes et al. 2018). In a clinical trial, high‐dose EGCG (856.8 mg/day) significantly reduced ghrelin levels while elevating adiponectin, correlating with weight loss in women with central obesity (Chen et al. 2016). Capsaicin enhances satiety and reduces energy intake through activation of TRPV1 channels in the gastrointestinal tract and central nervous system (Christie et al. 2018).

### 3.6 Oxidative Stress and Inflammation Attenuation

Natural products exhibit potent antioxidant and anti‐inflammatory properties (Aziz et al. 2023). Polyphenols scavenge free radicals directly through electron donation while also upregulating endogenous antioxidant systems (Aranaz et al. 2019; Zahran et al. 2025). Curcumin activates nuclear factor erythroid 2‐related factor 2 (Nrf2), a master regulator of antioxidant response element (ARE), driven genes encoding superoxide dismutase (SOD), catalase, glutathione peroxidase (GPx), and heme oxygenase‐1 (HO‐1) (Abd El‐Hameed et al. 2021; Ghareghomi et al. 2021).

Resveratrol attenuates adipose tissue inflammation by inhibiting NF‐κB activation and reducing pro‐inflammatory cytokine expression (Tumor Necrosis Factor Alpha [TNF‐α], Interleukin‐6 [IL‐6], Interleukin‐1β [IL‐1β]). In HFD‐fed mice, resveratrol supplementation reduces macrophage infiltration into adipose tissue and promotes polarization from pro‐inflammatory M1 to anti‐inflammatory M2 phenotype (Andrade et al. 2014). Quercetin decreases oxidative stress markers (malondialdehyde, protein carbonyls) and inflammatory mediators in brown adipose tissue, protecting thermogenic capacity (Boots et al. 2011).

Berberine exerts anti‐inflammatory effects through multiple mechanisms, including suppression of nuclear factor‐kappa B (NF‐κB) and NLRP3 inflammasome activation, reducing IL‐1β and Interleukin‐18 (IL‐18) production (Sun et al. 2024). In adipose tissue macrophages, berberine promotes M2 polarization through AMPK‐dependent autophagy activation, mitigating obesity‐induced inflammation (Han et al. 2020). These anti‐inflammatory and antioxidant effects contribute to improved insulin sensitivity and metabolic health beyond simple weight reduction (Golbidi et al. 2012; Nurkolis et al. 2025).

### 3.7 Mechanistic Convergence and Pathway Responsiveness in Advanced Obesity

Multiple natural products converge on the modulation of AMP‐activated protein kinase (AMPK) and peroxisome proliferator‐activated receptor gamma (PPARγ), raising important questions regarding pathway responsiveness in advanced obesity. Unlike leptin signaling, which exhibits well‐characterized central and peripheral resistance during obesity progression, AMPK and PPARγ do not develop classical ligand resistance. However, accumulating evidence indicates that their downstream signaling efficiency may be attenuated in advanced obesity due to chronic low‐grade inflammation, ectopic lipid accumulation, mitochondrial dysfunction, and altered co‐regulator availability (Chandrasekaran and Weiskirchen 2024; Saltiel and Olefsky 2017).

Preclinical and clinical data suggest that AMPK activation remains pharmacologically achievable in obese states, but the magnitude of metabolic benefit, such as enhancement of fatty acid oxidation and improvement of insulin sensitivity, is often reduced compared with lean or early‐stage obesity models (Ruderman et al. 2013). Similarly, PPARγ remains ligand‐responsive; however, its transcriptional output may be functionally altered by inflammatory signaling pathways, post‐translational modifications, and adipose tissue dysfunction characteristic of severe obesity, rather than true receptor resistance (Ahmadian et al. 2013; Tontonoz and Spiegelman 2008).

Importantly, current evidence does not support the development of leptin‐like absolute resistance to AMPK or PPARγ modulation. Instead, these pathways appear to retain conditional pharmacological targetability, particularly when interventions are implemented in early‐to‐moderate obesity or combined with strategies that reduce inflammatory burden, improve insulin sensitivity, or enhance metabolic flexibility. This may partly explain the variability observed across clinical trials of natural products targeting these pathways.

## 4 Notable Compounds and Herbal Sources

### 4.1 Polyphenols

#### 4.1.1 Catechins (Green Tea)

Green tea ( *Camellia sinensis* ) catechins, particularly (−)‐epigallocatechin‐3‐gallate (EGCG), represent the most extensively studied polyphenolic compounds for obesity management (Capasso et al. 2025; Nagle et al. 2006). Green tea contains four major catechins: EGCG (50%–80% of total catechins), (−)‐epicatechin gallate (ECG), (−)‐epigallocatechin (EGC), and (−)‐epicatechin (EC) (Tian et al. 2021). EGCG exhibits multiple anti‐obesity mechanisms: inhibition of intestinal lipid absorption through lipase inhibition and interference with micelle formation, suppression of adipogenesis via downregulation of PPARγ and C/EBPα, activation of AMPK promoting fatty acid oxidation, and enhancement of energy expenditure through COMT inhibition (Churm et al. 2023; Kim and Jang 2014).

Preclinical studies have demonstrated consistent anti‐obesity effects of EGCG in HFD‐induced obesity models. In C57BL/6 mice fed HFD supplemented with EGCG (50–100 mg/kg/day), body weight gain, adipose tissue mass, hepatic steatosis, and serum lipids were significantly reduced compared to controls (Chen et al. 2011). These effects were accompanied by enhanced autophagy and lipolysis in white adipose tissue, increased expression of genes involved in fatty acid oxidation (CPT‐1, ACO, MCAD, PPARα), and suppression of lipogenic genes (FAS, ACC, SREBP‐1c) (Li et al. 2018).

Clinical trials have yielded promising but variable results. A meta‐analysis of randomized controlled trials found that green tea catechin supplementation (containing 100–460 mg EGCG) resulted in mean weight loss of 1.31 kg (95% CI: −2.05 to −0.56) greater than placebo over 12 weeks (Rondanelli and Riva 2021). A well‐designed double‐blind trial in 102 women with central obesity demonstrated that high‐dose EGCG (856.8 mg/day) for 12 weeks produced significant reductions in body weight (−1.1 kg), BMI, and waist circumference compared to placebo, with good tolerability and no adverse events (Chen et al. 2016). However, some studies have failed to demonstrate significant effects, possibly due to variations in EGCG dosage, formulation, study population, and genetic polymorphisms in COMT affecting individual responsiveness (Yan and Cao 2025).

#### 4.1.2 Curcumin

Curcumin, the principal curcuminoid from turmeric ( *Curcuma longa* ) rhizomes, exhibits diverse pharmacological properties including anti‐inflammatory, antioxidant, and metabolic regulatory effects (Metawea et al. 2023) (89012456789). Curcumin inhibits adipogenesis through multiple mechanisms: downregulation of PPARγ, C/EBPα, and Sterol Regulatory Element‐binding Protein‐1 (SREBP‐1c); activation of Wnt/β‐catenin signaling maintaining preadipocytes in undifferentiated state; suppression of angiogenesis in adipose tissue; and modulation of adipokine secretion (Ahn et al. 2010; Wu et al. 2019).

In 3T3‐L1 adipocytes, curcumin dose‐dependently inhibits differentiation, reduces triglyceride accumulation, and decreases expression of adipogenic markers (aP2, adiponectin, FAS) (Ejaz et al. 2009). HFD‐fed rodents supplemented with curcumin (50–100 mg/kg) exhibit reduced body weight gain (5%–10%), decreased adipose tissue mass, improved glucose tolerance, and enhanced insulin sensitivity (Koboziev et al. 2020). Curcumin also attenuates hepatic steatosis by activating AMPK, inhibiting lipogenesis, and promoting fatty acid β‐oxidation (Guariglia and Saba 2023).

Despite compelling preclinical evidence, clinical translation has been hampered by curcumin's poor bioavailability, rapid metabolism, and low plasma concentrations achieved with oral administration (Jabur et al. 2025). Novel formulations employing nanoparticles (Metawea et al. 2023), phytosomes (Talebi et al. 2025), and piperine co‐administration (Hosseini et al. 2025) have demonstrated improved bioavailability. Limited clinical trials suggest modest benefits: a 12‐week study in overweight/obese subjects found that curcumin supplementation (1 g/day) produced small but significant reductions in BMI and waist circumference (Alsharif and Almuhtadi 2021). Larger, well‐designed clinical trials are needed to establish clinical efficacy and optimal dosing strategies.

#### 4.1.3 Resveratrol

Resveratrol, a stilbene polyphenol abundant in grapes, red wine, berries, and peanuts, has gained prominence for its anti‐obesity and metabolic benefits (Benbouguerra et al. 2021). Resveratrol activates SIRT1, an NAD^+^‐dependent deacetylase that regulates energy metabolism, mitochondrial biogenesis, and adipose tissue biology (Huang et al. 2021; Li et al. 2013). SIRT1 activation inhibits PPARγ activity through deacetylation, suppressing adipogenesis and promoting lipolysis (Mayoral et al. 2015). Additionally, resveratrol activates AMPK, enhances mitochondrial function, and promotes the browning of white adipose tissue through upregulation of UCP1, PGC‐1α, and PRDM16 (Terzo et al. 2024).

Mechanistic studies have revealed that resveratrol's anti‐obesity effects involve modulation of gut microbiota. In HFD‐fed mice, resveratrol supplementation increases beneficial Lactobacillus and Bifidobacterium species while reducing pathogenic Proteobacteria, correlating with improved metabolic parameters (Wang et al. 2020). Resveratrol also enhances intestinal barrier integrity, reduces LPS translocation, and attenuates systemic inflammation (Yu et al. 2024).

Clinical evidence for resveratrol's anti‐obesity effects remains limited and inconsistent (Hillsley et al. 2022). Some trials have demonstrated improvements in insulin sensitivity and inflammatory markers without significant weight loss. A systematic review concluded that resveratrol supplementation produces modest metabolic benefits in obese/overweight individuals but lacks robust evidence for substantial weight reduction (Christenson et al. 2016). Ongoing clinical trials are investigating higher doses and novel formulations to enhance bioavailability and clinical efficacy.

### 4.2 Alkaloids

#### 4.2.1 Capsaicin

Capsaicin, the pungent alkaloid from Capsicum species (chili peppers), exerts potent thermogenic and anti‐obesity effects (Zheng et al. 2017). Capsaicin activates TRPV1 cation channels expressed in sensory neurons, adipocytes, and hypothalamus, triggering calcium influx and downstream signaling cascades (Wang et al. 2025). In brown adipose tissue, TRPV1 activation enhances UCP1‐dependent thermogenesis through sympathetic nervous system activation and direct cellular mechanisms (Wang et al. 2025). Remarkably, capsaicin induces browning of white adipose tissue, converting energy‐storing white adipocytes into thermogenic beige adipocytes (Baskaran et al. 2016).

At the molecular level, capsaicin activates AMPK, calcium/calmodulin‐dependent protein kinase II (CaMKII), and p38 MAPK in adipocytes, leading to upregulation of PGC‐1α, UCP1, PRDM16, and bone morphogenetic protein 8b (BMP8b), key regulators of thermogenic programming (Abdillah and Yun 2024; Wang, Wang, and Hu 2023). In HFD‐fed mice, dietary capsaicin (0.01% *w*/*w*) prevents obesity, reduces adiposity, improves glucose tolerance, and enhances whole‐body energy expenditure through browning of subcutaneous adipose tissue (Shen et al. 2017).

Capsaicin also suppresses appetite and reduces food intake through multiple mechanisms, including activation of TRPV1 channels in the gastrointestinal tract, modulation of satiety hormone secretion (CCK, GLP‐1), and effects on hypothalamic feeding circuits (Wang et al. 2025). Human studies have demonstrated that capsaicin supplementation (2.56–10 mg/meal) increases energy expenditure, enhances fat oxidation, and reduces appetite, contributing to modest weight loss (Janssens et al. 2013).

#### 4.2.2 Berberine

Berberine, an isoquinoline alkaloid from *Berberis vulgaris* , Coptis chinensis, and other medicinal plants, exhibits remarkable anti‐obesity and metabolic regulatory effects (Zieniuk and Pawełkowicz 2025). Berberine activates AMPK through inhibition of mitochondrial complex I, creating a cellular energy deficit that triggers AMPK phosphorylation (Hu et al. 2024). AMPK activation leads to suppression of lipogenic enzymes (ACC, FAS, SREBP‐1c), enhancement of fatty acid oxidation (CPT‐1, ACO), and inhibition of adipogenesis through downregulation of PPARγ and C/EBPα (Cai et al. 2025; Hong et al. 2023).

Beyond direct metabolic effects, berberine profoundly modulates gut microbiota composition (He et al. 2022). In obese rodents, berberine supplementation increases beneficial bacteria ( *Akkermansia muciniphila* , *Bacteroides*, *Lactobacillus*) while reducing pro‐inflammatory species, leading to enhanced intestinal barrier function, reduced endotoxemia, and attenuated systemic inflammation (Zhu et al. 2018). Berberine also promotes M2 macrophage polarization in adipose tissue, mitigating obesity‐induced inflammation (Han et al. 2020).

Clinical evidence supports berberine's therapeutic potential for obesity and metabolic syndrome. A meta‐analysis of randomized controlled trials found that berberine supplementation (500–1500 mg/day for 8–24 weeks) significantly reduced body weight (weighted mean difference: −2.05 kg), BMI (−1.04 kg/m^2^), waist circumference, and triglycerides compared to placebo or lifestyle intervention alone (Asbaghi et al. 2020). Berberine also improved glycemic control and insulin sensitivity in patients with type 2 diabetes and metabolic syndrome (Yin et al. 2008). Side effects are generally mild, primarily consisting of gastrointestinal disturbances (diarrhea, constipation, abdominal pain) that diminish with continued use (Liu, Zhao, et al. 2025).

### 4.3 Terpenoids and Carotenoids

Carotenoids, tetraterpene pigments responsible for yellow, orange, and red colors in plants, exhibit anti‐obesity properties through diverse mechanisms (Maoka 2020). β‐Carotene, a provitamin A carotenoid, activates the SIRT1/AMPK pathway, decreasing hepatic lipogenesis and reducing IL‐6 expression in mice (El‐Marasy et al. 2024). β‐Cryptoxanthin, another provitamin A carotenoid from mandarin oranges, induces UCP1 expression in beige adipocytes via the retinoic acid receptor (RAR) pathway, promoting thermogenesis in HFD‐fed mice (Benbaibeche et al. 2025).

Fucoxanthin, a unique marine carotenoid from brown seaweeds (Undaria pinnatifida, Laminaria japonica), demonstrates potent anti‐obesity effects (Ding et al. 2025). Fucoxanthin increases energy expenditure through upregulation of UCP1 in white adipose tissue and enhancement of mitochondrial biogenesis via PGC‐1α activation (Wu et al. 2014). In obese rodents, fucoxanthin supplementation (0.1%–0.2% of diet) reduces body weight, visceral fat, and hepatic lipids while improving insulin sensitivity (Beppu et al. 2012). Human clinical trials are limited but suggest potential benefits for body composition improvements (Kumarasinghe and Gunathilaka 2024).

Lycopene, the red pigment in tomatoes, transactivates PPARγ and modulates inflammatory signaling through NF‐κB inhibition, reducing adipose tissue inflammation in obesity (Zhu et al. 2020). Astaxanthin, a red carotenoid from salmon and microalgae, activates PPARα while inhibiting PPARγ and Akt activity, reducing hepatic lipogenesis and inflammation in HFD‐induced obesity (Jia et al. 2016).

### 4.4 Saponins

Saponins are glycosidic compounds characterized by triterpene or steroid aglycone backbones attached to sugar moieties (Moses et al. 2014). These amphipathic molecules exhibit anti‐obesity effects primarily through pancreatic lipase inhibition, reduced dietary fat absorption, and modulation of lipid metabolism (Subramaniyan and Hanim 2025). Ginsenosides from *Panax ginseng* inhibit adipogenesis, reduce adipocyte size, and improve insulin sensitivity in preclinical models (Gao et al. 2013). *Platycodon grandiflorum* saponins regulate multiple signaling pathways including PI3K‐Akt, JAK–STAT, and MAPK, contributing to anti‐obesity effects (Han and Luo 2024).

In vitro studies demonstrate that saponins from various sources inhibit pancreatic lipase with IC₅₀ values ranging from 0.5 to 50 μg/mL, comparable to or exceeding orlistat's potency (Marrelli et al. 2016). Saponin‐rich extracts reduce postprandial lipemia, increase fecal lipid excretion, and prevent diet‐induced obesity in rodent models (Han et al. 2001).

## 5 In Vivo and Clinical Evidence

### 5.1 Preclinical Studies

Preclinical research using rodent models of diet‐induced obesity has provided extensive evidence for the anti‐obesity efficacy of natural products (Martins et al. 2022). High‐fat diet (HFD) models, typically consisting of 45%–60% calories from fat, reliably induce obesity, insulin resistance, hepatic steatosis, and metabolic dysfunction resembling human obesity (de Moura et al. 2021). Natural product interventions in these models have demonstrated significant improvements across multiple parameters (Benbaibeche et al. 2025).

Green tea catechins and EGCG have been most extensively studied in preclinical models (Singh et al. 2011). In C57BL/6 mice, EGCG supplementation (0.2%–0.5% of diet or 50–100 mg/kg body weight) for 8–20 weeks reduces body weight gain by 10%–15%, decreases adipose tissue mass by 20%–40%, and improves glucose tolerance and insulin sensitivity (Sae‐tan et al. 2011). Mechanistic investigations reveal that EGCG enhances fatty acid oxidation in adipose tissue, liver, and skeletal muscle through AMPK activation while suppressing lipogenic gene expression (Zhang, Xie, et al. 2024).

Berberine demonstrates remarkable efficacy in preventing and reversing obesity in rodent models (Ilyas et al. 2020). In HFD‐fed mice and rats, berberine (50–200 mg/kg body weight) reduces body weight by 8%–12%, decreases visceral adiposity, improves hepatic steatosis, and enhances insulin sensitivity (Kong, Yang, Nie, Zhang, et al. 2025). These effects are mediated through AMPK activation, modulation of gut microbiota, reduction in adipose tissue macrophage infiltration, and promotion of browning in white adipose tissue (Ilyas et al. 2020; Kong, Yang, Nie, Zhang, et al. 2025).

Combination approaches have shown promise in preclinical studies. A composition of nine anti‐adipogenic phytonutrients improved glucose tolerance and reduced weight gain, liver steatosis, visceral adiposity, and inflammatory markers more effectively than individual compounds in obese mice (Urasaki and Le 2022). This suggests potential synergistic or complementary effects of combining natural products with different mechanisms of action.

However, a major limitation in translating preclinical findings on natural products into clinical practice lies in the frequent use of supraphysiological doses in animal models (Wei et al. 2025). Many experimental studies employ doses of compounds such as epigallocatechin‐3‐gallate (EGCG; 50–100 mg/kg) or berberine (100–200 mg/kg) that substantially exceed human‐equivalent doses when scaled using body surface area conversion. While such dosing strategies may be appropriate for mechanistic proof‐of‐concept studies, they raise important concerns regarding physiological plausibility and clinical relevance.

Moreover, interspecies differences in absorption, metabolism, tissue distribution, and elimination further complicate dose extrapolation. For instance, rodents often exhibit faster clearance rates and lower oral bioavailability for polyphenols compared with humans, potentially necessitating higher experimental doses to achieve detectable biological effects. However, reliance on high‐dose paradigms may overestimate efficacy and underestimate safety risks in humans (Marshall et al. 2023).

Importantly, human clinical trials typically utilize substantially lower doses of these compounds, often yielding more modest but physiologically relevant effects. Therefore, preclinical efficacy should be interpreted cautiously and viewed primarily as evidence of biological potential rather than direct therapeutic equivalence (Marshall et al. 2023; Wei et al. 2025). Future studies should prioritize pharmacokinetic‐guided dosing, clinically achievable exposure levels, and formulation strategies that enhance bioavailability to improve translational validity.

### 5.2 Human Clinical Trials

Importantly, not all mechanistic pathways described in preclinical models translate uniformly into clinically meaningful outcomes. Among the multiple molecular targets proposed for natural products, activation of AMP‐activated protein kinase (AMPK) and modulation of insulin sensitivity emerge as the most consistently linked to human clinical benefits. For instance, compounds such as berberine and green tea catechins demonstrate AMPK activation in experimental models, which aligns with observed improvements in glycaemic control and lipid profiles reported in randomized clinical trials. In contrast, several additional pathways frequently cited in preclinical studies, including Wnt/β‐catenin and mTOR signaling, currently lack sufficient human evidence to support their clinical relevance in obesity management.

A systematic review and meta‐analysis of 279 clinical trials evaluating herbal medicines for obesity found that several interventions demonstrated significant but modest effects. Green tea extracts produced mean weight loss of 1.31 kg (95% CI: −2.05 to −0.56) greater than placebo, with pooled BMI reduction of 0.55 kg/m^2^. The magnitude of effect appears dose‐dependent, with higher EGCG doses (> 500 mg/day) yielding superior results (Payab et al. 2020).

Garcinia cambogia extracts containing hydroxy citric acid have shown inconsistent results in clinical trials. Some studies reported significant weight loss (2–4 kg over 8–12 weeks), while others found no difference compared to placebo. A meta‐analysis concluded that while Garcinia cambogia produced statistically significant weight loss, the magnitude was small (approximately 0.88 kg) and unlikely to be clinically meaningful (Onakpoya et al. 2011).

Cinnamon ( *Cinnamomum verum* ) supplementation has demonstrated modest benefits in metabolic parameters (Bibi et al. 2024). Clinical trials in patients with type 2 diabetes and metabolic syndrome found that cinnamon (1–6 g/day for 8–12 weeks) significantly reduced bodyweight, BMI, waist circumference, fasting glucose, and insulin resistance (Roussel et al. 2009).

Saffron ( *Crocus sativus* ) extracts have shown promise for appetite suppression and mood improvement in overweight individuals (Mashmoul et al. 2013). A randomized controlled trial found that Satiereal (176.5 mg/day of saffron stigma extract) for 8 weeks significantly reduced snacking frequency and promoted weight loss in mildly overweight women (Gout et al. 2010). Another study in coronary artery disease patients demonstrated that saffron aqueous extract significantly reduced BMI, waist circumference, fat mass, and appetite compared to placebo (Abedimanesh et al. 2017).

From a clinical perspective, the magnitude, durability, and safety of therapeutic effects are more informative than mechanistic diversity alone, as shown in Table 1. Although several natural products demonstrate statistically significant effects on body weight or metabolic parameters, the absolute effect sizes are generally modest when compared with standard pharmacotherapy. Consequently, the clinical relevance of these interventions should be interpreted within the context of adjunctive or supportive strategies rather than as standalone treatments for obesity, particularly in individuals with advanced disease or multiple comorbidities.

**TABLE 1:** Summary of key human clinical trials of natural products in obesity management.

Compound	Study (author, year)	Study design	Sample size (*n*)	Dose/formulation	Duration	Main outcomes	Adverse events	Trial quality (risk of bias)
Green tea catechins (EGCG)	Hursel et al. (2014)	Randomized, double‐blind, placebo‐controlled	240	EGCG 300–800 mg/day	12 weeks	Modest reduction in body weight (1–1.5 kg); improved fat oxidation	Mild GI discomfort; rare nausea	Low risk
Green tea extract	Chen et al. (2016)	Randomized controlled trial	182	Standardized green tea extract	12 weeks	Significant decrease in BMI and waist circumference	No serious adverse effects reported	Some concerns
Berberine	Yin et al. (2008)	Randomized controlled trial	116	Berberine 500 mg × 3/day	12 weeks	Reduced body weight, fasting glucose, HbA1c	Mild diarrhea and abdominal discomfort	Moderate risk
Saffron ( *Crocus sativus* )	Gout et al. (2010)	Randomized, double‐blind trial	60	Saffron extract 176 mg/day	8 weeks	Reduced snacking frequency and appetite; minor weight loss	Well tolerated	Some concerns
Cinnamon	Roussel et al. (2009)	Randomized controlled trial	58	Cinnamon 2 g/day	12 weeks	Modest reduction in fasting glucose; inconsistent weight effects	None significant	Moderate‐high risk

To complement the clinical trial evidence, a concise summary of the major meta‐analyses and systematic reviews examining natural products in obesity management is provided in Table 2. These studies offer higher‐level evidence that integrates multiple trials, enhancing interpretability and highlighting consistency, heterogeneity, and overall effect sizes.

**TABLE 2:** Summary of major meta‐analyses and systematic reviews on natural products for obesity management.

Compound/outcome	Citation	No. of trials	Pooled effect	95% CI	Heterogeneity/comments	Adverse events
Green tea catechins (EGCG)/weight loss	Ilyas et al. (2020)	Pooled RCTs	−1.31 kg (favoring catechins)	−2.05 to −0.56	Higher EGCG (> 500 mg/day) show superior results; heterogeneity across trials	Mostly mild GI symptoms at moderate doses; hepatotoxicity rare but associated with high‐dose supplements
Berberine/metabolic outcomes	Liu, Zhao, et al. (2025)	Multiple RCTs	Significant improvements in FPG/HbA1c and lipids; −2.05 kg (body weight). −1.04 kg/m^2^ (BMI)	−0.847 to −0.183	Many trials small, variable in region (several from China), heterogeneity present	GI side effects common; drug interaction cautions
Garcinia cambogia (HCA)/weight loss	Onakpoya et al. (2011)	12 RCTs	−0.88 kg (favoring Garcinia)	−1.75 to −0.00	High heterogeneity and variable trial quality	GI symptoms reported; concerns about product quality/adulteration in market

GRADE principles were applied qualitatively to summarize the certainty of evidence for major clinical claims. Downgrading decisions were standardized across compounds and based on predefined criteria. Consistency was judged by the agreement in direction and magnitude of effects across available randomized trials and meta‐analyses, with unexplained heterogeneity leading to downgrading. Precision was evaluated based on reported effect sizes, stability of estimates across studies, and the width of confidence intervals where available, with small or variable effects considered imprecise. Publication bias was assessed indirectly through bias evaluations reported in existing meta‐analyses, the predominance of small positive trials, and the limited availability of large, independently funded studies. As this was a narrative review, formal quantitative GRADE scoring was not performed; however, uniform qualitative criteria were applied to ensure internal consistency across interventions. This summary reflects major claims, grade certainty, and rationale for grading across the human clinical trials included in this narrative review (Table 3).

**TABLE 3:** Grade summary of evidence for major clinical outcomes of natural products in obesity.

Intervention	Major claim	GRADE certainty	Rationale for grading
Berberine	Improvement in glycemic control (HbA1c) and lipids	Moderate	(+) Strong magnitude of effect; plausible mechanism (AMPK). (−) Risk of bias from unblinding (GI side effects); publication bias
Berberine	Weight loss (−2.05 kg)	Low	(−) Side effects may confound appetite/intake; effect size is modest
Green Tea (EGCG)	Weight loss (−1.31 kg)	Very Low	(−) High inconsistency (genetic variance); Risk of bias (unblinding); effect size is small and variable
Curcumin	Reduced adiposity	Very Low	(−) Critical inconsistency due to formulation differences; bioavailability limits generic recommendations
Capsaicin	Increased energy expenditure/weight loss	Very Low	(−) Critical risk of bias (impossible to blind); Reliance on surrogate markers (acute thermogenesis)
Garcinia Cambogia	Weight loss (~0.88 kg)	Low	(−) Effect size is statistically significant but clinically marginal; bias in outcome assessment likely drives the small effect

### 5.3 Sources of Heterogeneity and Null/Negative Clinical Trials

While numerous clinical trials and meta‐analyses support modest efficacy of natural products such as EGCG, berberine, saffron, and Garcinia for obesity management, variability in study outcomes is notable. Several factors contribute to this heterogeneity. Differences in dose and formulation (e.g., standard vs. non‐standardized extracts, phytosome or nanoparticle formulations for curcumin) can substantially affect bioavailability and clinical efficacy (Hursel et al. 2014; Mahdi et al. 2025). Population characteristics, including baseline BMI, age, sex, ethnicity, and comorbidities, influence responsiveness; for example, low‐activity COMT genotypes show greater weight loss with green tea catechins (Hursel et al. 2014), and gut microbiota composition modulates polyphenol bioavailability (Mahdi et al. 2025).

Some trials failed to demonstrate significant weight loss or metabolic improvement (e.g., certain low‐dose EGCG or Garcinia studies) due to insufficient duration, small sample size, or inadequate standardization of the intervention (Chen et al. 2016; Onakpoya et al. 2011). These null or negative trials highlight the need for careful dose selection, standardized formulations, and adequately powered studies.

Funnel‐plot analyses reported in meta‐analyses of EGCG and berberine trials suggest a moderate risk of bias favoring positive studies, with smaller negative trials often unpublished (Liu, Zhao, et al. 2025; Rondanelli and Riva 2021). Awareness of these factors is critical for interpretation of clinical results and for guiding future trial design.

### 5.4 Critical Analysis of Study Quality and Limitations

Critical evaluation of clinical trial quality reveals several methodological limitations affecting interpretation and generalizability. Many studies suffer from small sample sizes (< 50 participants), short intervention durations (< 12 weeks), inadequate randomization and blinding procedures, high dropout rates, and insufficient statistical power. A quality assessment using Jadad, Delphi, and Cochrane criteria found that common deficiencies included non‐blinding of outcome assessors (82% of studies), inadequate reporting of patient compliance (65%), missing drop‐out/withdrawal procedures (58%), and inappropriate baseline characteristics (45%) (Ahmad et al. 2021).

Also, a key limitation of the current clinical literature is that most trials evaluating natural products in obesity employ placebo‐controlled designs rather than active comparators such as approved anti‐obesity or antidiabetic pharmacotherapies. While placebo‐controlled trials are appropriate for establishing biological activity and safety, they do not permit direct conclusions regarding comparative efficacy. Consequently, observed effects should be interpreted as indicative of adjunctive or supportive benefits rather than as alternatives to established pharmacological treatments. From a clinical perspective, this suggests that natural products may have greater relevance in early‐stage obesity, in metabolically mild phenotypes, or as complementary interventions alongside lifestyle modification or standard drug therapy. Head‐to‐head trials and add‐on designs comparing natural products with approved agents are needed to define their true clinical utility and positioning (Gadde and Atkins 2020).

Furthermore, publication bias favoring positive results likely inflates the apparent efficacy of natural products. Heterogeneity in study populations (age, ethnicity, baseline BMI, comorbidities), intervention characteristics (extract standardization, dosage, formulation), and outcome measures complicates meta‐analysis and cross‐study comparisons (Peter and Nagel 2017). The lack of long‐term studies (> 1 year) limits understanding of sustained efficacy and safety (Cuzick 2023).

Genetic polymorphisms influencing natural product metabolism and response represent an underappreciated source of variability (Abdulghani and Al‐Fayyadh 2024). For example, COMT polymorphisms affect individual responsiveness to green tea catechins, with low‐activity COMT genotypes exhibiting greater weight loss (Hursel et al. 2014). Gut microbiota composition also modulates bioavailability and efficacy of polyphenols and other phytochemicals (Mahdi et al. 2025). Future precision nutrition approaches incorporating pharmacogenomics and microbiome profiling may enhance clinical efficacy and personalization.

Although this article is a narrative review and not a systematic review, a qualitative appraisal of the methodological rigor of the key human clinical trials cited was conducted to enhance interpretability of the evidence. The assessment was based on core Cochrane risk‐of‐bias domains (randomization, allocation concealment, blinding, incomplete outcome data, and selective reporting) as reported in the original studies. A summary of this evaluation is presented in Table 4.

**TABLE 4:** Risk‐of‐bias assessment of key clinical trials (Cochrane‐based qualitative evaluation).

Compound	Randomization	Allocation concealment	Blinding	Incomplete outcome data	Selective reporting	Overall risk of bias
EGCG/green tea	Low	Some concerns	Low	Some concerns	Low	Some concerns
Berberine	Some concerns	Some concerns	Some concerns	Some concerns	Some concerns	Moderate risk
Saffron	Low	Low	Low	Some concerns	Low	Some concerns
Cinnamon	Some concerns	Unclear	Some concerns	Some concerns	Some concerns	Moderate–High risk
Garcinia cambogia	Some concerns	Unclear	Some concerns	Some concerns	Some concerns	High risk
Capsaicin	Some concerns	Some concerns	Some concerns	Some concerns	Low	Moderate risk

To strengthen future research, well‐designed randomized controlled trials with larger, adequately powered cohorts and longer follow‐up periods are needed to evaluate both sustained efficacy and long‐term safety. Improved trial designs should incorporate standardized and chemically characterized formulations, clearly defined dosing regimens, and harmonized clinical endpoints, such as percentage weight loss, changes in body composition, and metabolic biomarkers. In addition, preregistered protocols, transparent reporting of negative or null findings, and intention‐to‐treat analyses would help reduce bias and enhance reproducibility.

## 6 Safety, Standardization, and Regulatory Considerations

Although natural products are frequently perceived as inherently safer than synthetic drugs, this assumption is not supported by pharmacological or clinical evidence. Safety is primarily determined by dose, formulation, duration of exposure, metabolic context, and potential drug‐herb interactions rather than by natural origin alone (Choi and Song 2021; Younes, Aggett, Aguilar, et al. 2018). Several bioactive phytochemicals demonstrate favorable safety profiles at dietary intake levels, yet may exhibit clinically relevant adverse effects when administered at pharmacological doses or in concentrated formulations.

### 6.1 Safety Profile and Adverse Effects

While natural products are generally perceived as safe, comprehensive safety assessment is essential for clinical application. The majority of phytochemicals exhibit favorable safety profiles at typical dietary and supplemental doses, but high‐dose or long‐term use may pose risks (Heydari et al. 2022), as shown in Tables 5 and 6.

**TABLE 5:** Safety profile and precautions of key natural products.

Compound	Common adverse effects	Dose‐related concerns	Key precautions
EGCG (green tea extract)	Mild GI symptoms	Hepatotoxicity reported with high‐dose or concentrated supplements	Avoid in liver disease; monitor liver enzymes during prolonged use
Berberine	Diarrhea, constipation, abdominal cramping	GI intolerance at higher doses	Caution with antidiabetic drugs; potential CYP450 interactions
Capsaicin	GI irritation, burning sensation	Gastric ulceration at high doses	Avoid excessive dosing; caution in peptic ulcer disease

**TABLE 6:** Cytochrome P450, P‐glycoprotein, and transporter interactions of major natural products.

Natural product	Enzyme/transporter affected	Direction of interaction	Potential clinical implications (polypharmacy context)	Level of evidence	Key references
Berberine	CYP3A4, CYP2D6, CYP2C9, P‐gp	Inhibition	Increased plasma levels of statins, antidiabetic drugs, calcium channel blockers; risk of hypoglycaemia when combined with antidiabetics	Human + in vitro	Guo et al. (2012); May and Schindler (2016)
Green Tea Extract (EGCG)	OATP1A2, OATP2B1, CYP3A4 (weak)	Inhibition	Reduced intestinal drug absorption; increased hepatotoxicity risk at high doses; altered exposure of β‐blockers and statins	Human	Chow et al. (2003); Younes, Aggett, Aguilar, et al. (2018)
Curcumin	CYP3A4, CYP2C9, CYP1A2, P‐gp	Inhibition	Altered clearance of anticoagulants, antidiabetics, and chemotherapeutics; clinical relevance depends on formulation	In vitro + limited human	Hegde et al. (2023); Volak et al. (2008)
Capsaicin	CYP1A2 (minor), TRPV1‐mediated pathways	Modulation	Minimal interaction at dietary doses; potential GI irritation may affect drug tolerance	Preclinical + limited human	Zhang, Zhang, et al. (2024)

Green tea extracts and EGCG have been extensively evaluated for safety (Younes, Aggett, Aguilar, et al. 2018). At moderate doses (< 800 mg EGCG/day), adverse events are rare and primarily limited to mild gastrointestinal symptoms (nausea, abdominal discomfort) (Chow et al. 2003). However, high‐dose EGCG supplements (> 1000 mg/day) have been associated with hepatotoxicity, including elevated liver enzymes, acute hepatitis, and rare cases of liver failure (Shi et al. 2021). The FDA issued warnings regarding hepatotoxicity risk with concentrated green tea extracts (Patel et al. 2013). Importantly, hepatotoxicity appears to be idiosyncratic, potentially related to genetic polymorphisms in UDP‐glucuronosyltransferase enzymes (Roth and Lee 2017). Regulatory agencies, including the European Food Safety Authority (EFSA) and the FDA, advise caution with concentrated green tea extracts and recommend monitoring liver function when exceeding standard doses (Younes, Aggett, Aguilar, et al. 2018).

Berberine's safety profile is generally favorable, with gastrointestinal side effects (diarrhea, constipation, abdominal cramping) being most common, occurring in approximately 10%–20% of users (Li and Wang 2023). These effects are usually mild and resolve with continued use or dose reduction (Ju et al. 2018). Berberine may interact with cytochrome P450 enzymes, potentially affecting the metabolism of concomitant medications (Guo et al. 2011). Caution is advised in patients taking antidiabetic medications due to potential additive hypoglycemic effects (May and Schindler 2016).

Capsaicin is generally regarded as safe (GRAS status) at culinary doses (Deshpande et al. 2016). Supplemental capsaicin may cause gastrointestinal irritation, burning sensations, and rarely, gastric ulceration at high doses (Xiang et al. 2022). Topical capsaicin preparations can cause skin irritation and should be used with caution (Hayman and Kam 2008).

Other natural compounds, including curcumin, saffron, and Garcinia extracts, generally exhibit low toxicity at typical supplemental doses (Alsharif and Almuhtadi 2021; Gout et al. 2010; Onakpoya et al. 2011). Nevertheless, monitoring for gastrointestinal discomfort and potential interactions with medications metabolized by CYP enzymes is recommended.

For clinical translation, natural products must comply with local and international regulatory guidelines. For instance, EFSA provides guidance on the use of botanical extracts in supplements, including dose thresholds and safety assessments (Marakis et al. 2018).

### 6.2 Quality Control and Standardization Challenges

Ensuring consistent quality, potency, and safety of herbal products represents a major challenge impacting clinical efficacy and regulatory approval (Wang, Chen, et al. 2023). Unlike synthetic pharmaceuticals with defined chemical structures, plant extracts contain complex mixtures of bioactive compounds whose composition varies based on botanical source, geographical origin, growing conditions, harvesting time, processing methods, and storage (Atanasov et al. 2015).

Standardization aims to establish consistent levels of marker compounds or bioactive constituents in herbal products (Srisittiratkul et al. 2025). For green tea extracts, standardization typically targets total catechin content and EGCG percentage (Younes, Aggett, Aguilar, et al. 2018). However, other constituents (caffeine, amino acids, and minerals) also contribute to biological activity, and their ratios may influence efficacy (Luo et al. 2024). WHO guidelines recommend multiple quality control parameters including macroscopic and microscopic authentication, physicochemical parameters (moisture content, ash value, extractive values), phytochemical profiling, and contaminant testing (Gempo et al. 2024).

Advanced analytical techniques have enhanced quality control capabilities. High‐performance liquid chromatography (HPLC), gas chromatography–mass spectrometry (GC–MS), and thin‐layer chromatography (TLC) enable quantification of marker compounds and detection of adulterants (Almoselhy et al. 2025). Metabolomic fingerprinting using nuclear magnetic resonance (NMR) or mass spectrometry provides comprehensive chemical profiles for authentication and batch‐to‐batch consistency monitoring (Riswanto and Windarsih 2022).

Adulteration with synthetic pharmaceuticals represents a serious safety concern, particularly in weight‐loss supplements (Jairoun et al. 2021). A systematic review found that 19%–46% of weight‐loss natural products contained undeclared pharmaceutical adulterants including sibutramine, phenolphthalein, and diuretics (Gheorghiu et al. 2025). Such adulteration poses significant health risks and undermines confidence in legitimate natural products.

### 6.3 Bioavailability Enhancement Strategies

Poor oral bioavailability limits the clinical efficacy of many phytochemicals (Hu et al. 2023). Factors contributing to low bioavailability include poor water solubility, extensive first‐pass metabolism, rapid elimination, and limited membrane permeability (Bhalani and Nutan 2022). Curcumin exemplifies this challenge, with oral bioavailability of < 1% due to poor aqueous solubility, extensive glucuronidation and sulfation, and rapid biliary excretion (Tabanelli and Brogi 2021).

Numerous strategies have been developed to enhance phytochemical bioavailability, as shown in Table 7. Co‐administration with piperine (black pepper alkaloid) inhibits glucuronidation and P‐glycoprotein‐mediated efflux, increasing curcumin bioavailability by 2000% (Di et al. 2015). Lipid‐based formulations (phytosomes, liposomes, nanoemulsions) improve solubility and absorption of lipophilic compounds (Rehman et al. 2024). Nanoparticle encapsulation protects compounds from degradation, enhances membrane permeability, and enables controlled release (Pugazhendhi et al. 2025).

**TABLE 7:** Clinical impact of formulation strategies on bioavailability and efficacy of natural products in obesity.

Compound	Formulation/strategy	Bioavailability improvement	Clinical endpoint observed	References
Curcumin	Phytosome	↑ plasma levels ~29‐fold	↓ CRP, improved metabolic biomarkers	Di Pierro et al. (2015)
Curcumin	Piperine co‐administration	↑ plasma levels ~20‐fold	Improved fasting glucose and HOMA‐IR in small trials	Panahi et al. (2017); Shoba et al. (1998)
Berberine	Nanoparticle or lipid‐based formulations	↑ systemic exposure (2–4 fold)	Improved FPG, HbA1c, and lipid profile	Zhang, Sheng, et al. (2020)
Berberine	Nanoemulsion	↑ oral bioavailability of BBR in rats by 212.02%	Blood glucose level of diabetic mice by NE was decreased by 3‐fold	Xu et al. (2019)

For catechins, strategies to improve bioavailability include structural modification (e.g., peracetylation), complexation with phospholipids, and nanoparticle delivery systems (Stoeva‐Grigorova and Ivanova 2025). Clinical studies have demonstrated that enhanced bioavailability formulations produce superior metabolic outcomes compared to conventional extracts at equivalent doses (Di Costanzo and Angelico 2019).

However, for many compounds, systematic clinical evidence linking enhanced bioavailability to improved clinical endpoints remains scarce. This represents an important research gap, emphasizing the need for well‐designed trials assessing not only pharmacokinetics but also clinical efficacy of optimized formulations.

### 6.4 Regulatory Framework and Approval Pathways

Regulatory frameworks for natural products vary substantially across jurisdictions, complicating international development and commercialization (Low et al. 2017). In the United States, most botanical products are regulated as dietary supplements under the Dietary Supplement Health and Education Act (DSHEA), which does not require pre‐market approval but restricts health claims (Wallace 2015). Products marketed as drugs must undergo FDA approval through the standard new drug application (NDA) process, requiring extensive preclinical and clinical data demonstrating safety and efficacy (van Norman 2016).

European regulation varies by country, with some nations (Germany, France) having well‐established systems for herbal medicinal products, while others regulate botanicals as food supplements (Gruenwald 2002). The European Medicines Agency (EMEA) provides guidelines for development of anti‐obesity drugs, requiring clinically meaningful weight reduction (≥ 5% placebo‐subtracted or > 10% baseline weight loss) after 1 year, accompanied by improvements in cardiovascular risk factors (Coutinho and Halpern 2024).

Traditional use registration pathways in Europe allow marketing of herbal medicines with documented traditional use (≥ 30 years, including ≥ 15 years in EU) without full clinical efficacy data (Jadhav et al. 2025). However, anti‐obesity indications are typically not eligible for traditional use registration due to safety concerns requiring robust clinical evidence.

China, India, and other Asian countries with rich traditional medicine systems have developed specific regulatory frameworks for traditional herbal products. In China, traditional Chinese medicines can be approved through simplified procedures if they meet standardization requirements and demonstrate acceptable safety. However, export to Western markets still requires compliance with FDA or EMA regulations (Chan Fung 2020).

The lack of harmonized global standards for natural products hinders clinical development, increases costs, and limits patient access to potentially beneficial therapies (Ng et al. 2022). Efforts toward international harmonization, such as WHO guidelines and International Conference on Harmonization (ICH) initiatives, represent important steps forward.

## 7 Future Perspectives and Challenges

### 7.1 Integration With Modern Therapeutic Approaches

The future of obesity management likely involves integrating natural products with conventional pharmacological and lifestyle interventions to achieve synergistic benefits (Chan et al. 2021). Combination strategies may enhance efficacy while minimizing adverse effects associated with high‐dose single agents (Halpern et al. 2010). For example, combining GLP‐1 receptor agonists with metabolic modulators like berberine or EGCG could provide complementary mechanisms: GLP‐1 agonists primarily affect appetite and glucose metabolism, while natural products modulate adipogenesis, inflammation, and gut microbiota (Kolhe and Manisha 2025).

Precision nutrition approaches incorporating pharmacogenomics, metabolomics, and microbiome profiling promise to optimize natural product selection and dosing for individual patients (Mansour et al. 2024). Identifying genetic variants affecting COMT activity could guide green tea catechin use, while microbiome signatures might predict responsiveness to polyphenol‐based interventions (Lai et al. 2019). Integration of wearable devices and digital health platforms could enable real‐time monitoring of metabolic responses, facilitating personalized optimization (Ghadi et al. 2025).

Natural products may also serve as adjuvants to bariatric surgery, potentially enhancing weight loss outcomes and preventing weight regain (Shaik Mohamed Sayed et al. 2023). Post‐surgical patients often experience nutritional deficiencies, inflammation, and metabolic changes that natural products with antioxidant and anti‐inflammatory properties could address (Nainu et al. 2023).

### 7.2 Novel Delivery Systems and Formulation Strategies

Innovative drug delivery systems are being established to overcome bioavailability limitations and enhance therapeutic efficacy (Ezike et al. 2023). Nanoparticle‐based delivery systems (polymeric nanoparticles, solid lipid nanoparticles, liposomes) protect phytochemicals from degradation, improve solubility, enhance cellular uptake, and enable targeted delivery to adipose tissue (Uti et al. 2025). Nano‐encapsulated EGCG demonstrates fourfold increased adipose tissue targeting compared to free EGCG (Das et al. 2025).

Microneedle patches represent an innovative transdermal delivery approach for anti‐obesity agents (Pan et al. 2025). Dissolving microneedles loaded with natural products or synthetic compounds penetrate the stratum corneum, delivering cargo directly into viable epidermis and dermis, bypassing first‐pass metabolism (Moawad et al. 2025). This technology shows promise for delivering compounds with poor oral bioavailability or rapid metabolism.

Stimuli‐responsive delivery systems that release cargo in response to specific physiological conditions (pH, temperature, enzymes) enable controlled, site‐specific delivery. For example, pH‐responsive nanoparticles could release payloads in the acidic environment of inflamed adipose tissue or the colon microenvironment (Wells et al. 2019).

### 7.3 Gut Microbiota as Therapeutic Target

The gut microbiota has emerged as a critical mediator of natural product anti‐obesity effects and a promising therapeutic target (Liu et al. 2021). Strategies to harness the gut microbiota include: (1) prebiotic approaches using poorly absorbed polyphenols to modulate microbial composition; (2) probiotic supplementation with beneficial strains ( *Akkermansia muciniphila* , *Lactobacillus*, *Bifidobacterium*); (3) synbiotic combinations of prebiotics and probiotics; and (4) postbiotic utilization of bacterial metabolites (SCFAs, secondary bile acids) (Reddy et al. 2024).

Engineered probiotics represent a cutting‐edge approach (Pesce and Seguella 2022). Genetically modified bacteria could be designed to produce anti‐obesity compounds (GLP‐1, AMPK activators, anti‐inflammatory molecules) directly in the gut, providing sustained local delivery (Wang et al. 2021; Ye et al. 2025). Safety and regulatory considerations for engineered probiotics require careful evaluation (Roe et al. 2022).

Fecal microbiota transplantation (FMT) has shown promising results in preclinical obesity models, with transplantation from lean donors improving metabolism in obese recipients (Zecheng et al. 2023). However, clinical trials in humans have yielded mixed results, and safety concerns regarding transmission of infectious agents and unknown metabolic consequences necessitate cautious development (Hanssen et al. 2021).

### 7.4 Sustainable Sourcing and Environmental Considerations

As demand for natural products increases, ensuring sustainable sourcing practices becomes imperative to prevent biodiversity loss and protect ecosystems. Many medicinal plants are threatened by overharvesting, habitat destruction, and climate change (Groner et al. 2022). The Convention on Biological Diversity and Nagoya Protocol provide frameworks for equitable sharing of benefits from genetic resources, but implementation remains challenging (Coolsaet et al. 2013).

Sustainable cultivation practices, including organic farming, agroforestry, and forest gardening, can meet demand while preserving ecosystems and supporting local communities (Robinson 2024). Biotechnological approaches such as plant cell culture, tissue culture, and metabolic engineering offer alternatives to wild harvesting (Mohaddab and El Goumi 2022). For example, cell culture systems can produce high‐value phytochemicals under controlled conditions, ensuring consistent quality and reducing environmental impact (Mohaddab and El Goumi 2022).

Marine‐derived compounds like fucoxanthin face unique sustainability challenges related to seaweed harvesting and aquaculture (Chadorshabi et al. 2025). Integrated multi‐trophic aquaculture systems, where seaweeds are cultivated alongside finfish and shellfish, provide environmentally sustainable production while contributing to ecosystem services (Ingle et al. 2022).

Climate change poses additional threats to medicinal plant availability and phytochemical composition. Rising temperatures, altered precipitation patterns, and extreme weather events affect plant distribution, growth, and secondary metabolite production (Alum 2024). Research on climate‐resilient cultivation practices and adaptive conservation strategies is essential.

### 7.5 Regulatory Challenges Across Different Global Regions

Despite growing scientific evidence supporting the potential role of natural products in obesity management, regulatory frameworks governing their development, approval, and clinical use vary substantially across global regions, posing significant barriers to translation. In the United States, natural products are primarily regulated as dietary supplements under the Dietary Supplement Health and Education Act (DSHEA), which does not require premarket demonstration of efficacy and places the burden of safety monitoring largely on post‐market surveillance (FDA 2013). While this framework facilitates market access, it also contributes to variability in product quality, standardization, and strength of clinical claims, complicating their integration into evidence‐based obesity treatment.

In contrast, regulatory authorities in the European Union apply more stringent requirements. The European Food Safety Authority (EFSA) mandates robust scientific substantiation for health claims, including well‐designed human intervention studies demonstrating clinically meaningful outcomes (EFSA 2012). As a result, many natural products with promising preclinical and early clinical data fail to meet the evidentiary threshold required for approved obesity‐related claims, highlighting a disconnect between emerging research and regulatory acceptance.

Regulatory approaches in Asia are similarly heterogeneous. In countries such as China, Japan, and South Korea, traditional herbal medicines are often regulated under separate frameworks that recognize historical use alongside modern scientific evidence (WHO 2013). While this allows greater flexibility in clinical application, challenges related to harmonization, quality control, and international recognition persist. In Japan, for example, Foods for Specified Health Uses (FOSHU) require clinical validation, yet the approval process remains lengthy and resource‐intensive (Iwatani and Yamamoto 2019).

In low‐ and middle‐income regions, including parts of Africa and Latin America, regulatory oversight of herbal and natural products is often limited by insufficient infrastructure, leading to inconsistent quality standards and reduced consumer protection (WHO 2019). These disparities underscore the need for globally harmonized guidelines addressing standardization, safety assessment, and evidence thresholds, particularly for conditions such as obesity that require long‐term intervention.

Future progress will depend on the development of adaptive regulatory frameworks that balance innovation with patient safety, encourage high‐quality clinical research, and accommodate regional differences in traditional medicine use. International collaboration among regulatory agencies, alongside alignment with evidence‐based evaluation criteria, will be critical for enabling the responsible integration of natural products into global obesity management strategies.

### 7.6 Addressing Research Gaps and Future Directions

Despite substantial progress, significant research gaps remain. Long‐term clinical trials (≥ 2 years) evaluating sustained efficacy, safety, and effects on hard clinical endpoints (cardiovascular events, mortality) are lacking for most natural products. Dose–response relationships, optimal formulations, and treatment durations require systematic investigation.

Mechanistic understanding at the molecular level continues to evolve. Advanced omics technologies (genomics, transcriptomics, proteomics, metabolomics, microbiomics) enable comprehensive characterization of natural product mechanisms, identifying biomarkers for response prediction and revealing novel therapeutic targets (Dai and Shen 2022; Saad 2023). Systems biology approaches integrating multi‐omics data can elucidate complex networks of interactions underlying anti‐obesity effects (Son et al. 2020).

The pediatric population, a critical demographic given rising childhood obesity rates, remains understudied (Quader et al. 2019). Most clinical trials exclude children, and safety and efficacy in developing individuals require specific evaluation (Field et al. 2004). Natural products may offer advantages for pediatric obesity given favorable safety profiles, but rigorous research is needed (Son 2024).

Special populations including pregnant and lactating women, elderly individuals, and those with comorbidities require targeted investigation (Grimsrud et al. 2015). Drug‐supplement interactions, particularly with medications for diabetes, hypertension, and cardiovascular disease commonly used in obese populations, need comprehensive characterization (Islam et al. 2024).

Finally, establishing clinically meaningful endpoints beyond simple weight loss is crucial. Obesity is a complex disease with multiple complications, and interventions should target improvements in metabolic health, cardiovascular risk factors, quality of life, and long‐term health outcomes. Composite endpoints incorporating weight loss, glycemic control, lipid profiles, inflammation markers, and patient‐reported outcomes would provide a more comprehensive efficacy assessment.

## 8 Conclusion

Obesity represents a complex, multifactorial global health crisis that demands innovative, multi‐targeted therapeutic approaches. Natural products derived from medicinal plants, fruits, vegetables, and other natural sources offer compelling advantages for obesity management, including diverse mechanisms of action, favorable safety profiles, and alignment with holistic health paradigms. The extensive body of preclinical evidence demonstrates that bioactive phytochemicals, particularly polyphenols like EGCG, curcumin, and resveratrol; alkaloids such as capsaicin and berberine; and other compounds including saponins, terpenoids, and carotenoids, exert significant anti‐obesity effects through modulation of lipid metabolism, inhibition of adipogenesis, enhancement of energy expenditure, regulation of appetite, modulation of gut microbiota, and attenuation of inflammation and oxidative stress.

Clinical translation has proven more challenging, with human trials yielding more modest and variable results compared to animal studies. Nonetheless, meta‐analyses and well‐designed randomized controlled trials support the therapeutic potential of select natural products, particularly green tea catechins, berberine, and certain herbal formulations, for producing clinically meaningful weight loss and metabolic improvements. The magnitude of effects, while modest compared to newer pharmaceutical agents like GLP‐1 receptor agonists, must be evaluated in the context of superior safety profiles, lower costs, and potential for long‐term adherence.

Critical challenges impeding broader clinical adoption include poor bioavailability of many phytochemicals, variability in product quality and standardization, limited long‐term safety data, inadequate understanding of optimal dosing and formulation strategies, and complex regulatory landscapes that vary across jurisdictions. Addressing these challenges requires concerted efforts involving: (1) development of enhanced delivery systems leveraging nanotechnology and novel formulations; (2) implementation of rigorous quality control measures and standardization protocols; (3) conduct of large‐scale, long‐term clinical trials with hard clinical endpoints; (4) investigation of combination therapies integrating natural products with conventional treatments; (5) adoption of precision nutrition approaches incorporating pharmacogenomics and microbiome profiling; and (6) establishment of sustainable sourcing practices protecting biodiversity and ecosystems.

The future of natural product‐based obesity therapeutics appears promising, with ongoing research elucidating molecular mechanisms, identifying novel bioactive compounds, optimizing delivery systems, and exploring synergistic combinations. Integration with modern pharmacological approaches, digital health technologies, and personalized medicine strategies may unlock the full therapeutic potential of these ancient remedies for contemporary health challenges. As the obesity epidemic continues its relentless trajectory, natural products represent an important, underutilized resource that warrants sustained scientific investigation, judicious clinical application, and thoughtful regulatory consideration. Through rigorous research, innovative development, and responsible stewardship, natural products can contribute meaningfully to the global fight against obesity and its devastating health, social, and economic consequences.

## Author Contributions

**Ohoud M. Marie:** conceptualized the review topic, designed the study framework, conducted the comprehensive literature search, analyzed and synthesized the data, and wrote the initial draft of the manuscript. **Abdelghafar Abu‐Elsaoud:** critical revision of the manuscript, and provided valuable scientific input to enhance the discussion and overall structure. Both authors reviewed and approved the final version of the manuscript before submission.

## Funding

This work was supported and funded by the Deanship of Scientific Research at Imam Mohammad Ibn Saud Islamic University (IMSIU) (grant number IMSIU‐DDRSP2602).

## Conflicts of Interest

The authors declare no conflicts of interest.

## Data Availability

The data that support the findings of this study are available from the corresponding author upon reasonable request.
